# Supplementary material for: Modelling DNA replication fork stability and collapse using chromatin fiber analysis and the R-ODD-BLOBS program
Source: bioRxiv. 2025 Nov 2:2024.11.01.621594. Preprint. [Version 2] doi: 10.1101/2024.11.01.621594 (PMC12636476; doi:10.1101/2024.11.01.621594)
Supplement: Supplement 1 [file media-1.pdf]

Modelling DNA replication fork stability and collapse using chromatin fiber analysis and the R-ODD-BLOBS program

SUPPLEMENTARY INFORMATION

AUTHORS:
Kerenza Cheng[1], Kazeera Aliar[2], Roozbeh Manshaei[3], Susan L Forsburg[4], Ali Mazalek[1,3], Sarah A Sabatinos[1,2, *]

AFFILIATIONS

1 – Molecular Science Graduate Program, Yeates School of Graduate and Postdoctoral Studies, Toronto Metropolitan University, Toronto ON M5B 2K3

2 – Department of Chemistry and Biology, Toronto Metropolitan University, Toronto ON M5B 2K3

3 – Synaesthetic Media Lab, The Creative School, Toronto Metropolitan University, Toronto ON M5B 2K3

4 – Department of Molecular and Computational Biology, University of Southern California, Los Angeles, California USA 90089

Supplemental Figure 1: Initial Scatter Plot of Channel intensities of One WT file
Supplemental Figure 2: Individual Replicates of BrdU Thresholding for Wt, *cds1Δ* and *mrc1Δ*.
Supplemental Figure 3: Individual Replicates of Rad51 Thresholding for Wt, *cds1Δ* and *mrc1Δ*.
Supplemental Figure 4: Individual Replicates of Cdc45 Thresholding for Wt, *cds1Δ* and *mrc1Δ*.
Supplemental Figure 5: Individual Replicates of BrdU Smoothing for Wt, *cds1Δ* and *mrc1Δ*.
Supplemental Figure 6: Individual Replicates of Rad51 Smoothing for Wt, *cds1Δ* and *mrc1Δ*.
Supplemental Figure 7: Individual Replicates of Cdc45 Smoothing for Wt, *cds1Δ* and *mrc1Δ*.
Supplemental Figure S8: Effects of Replication Fork Window size on Rad51 and Cdc45 colocalization

Supplemental Figure 1: Initial Scatter Plot of Channel intensities from a single wt image.

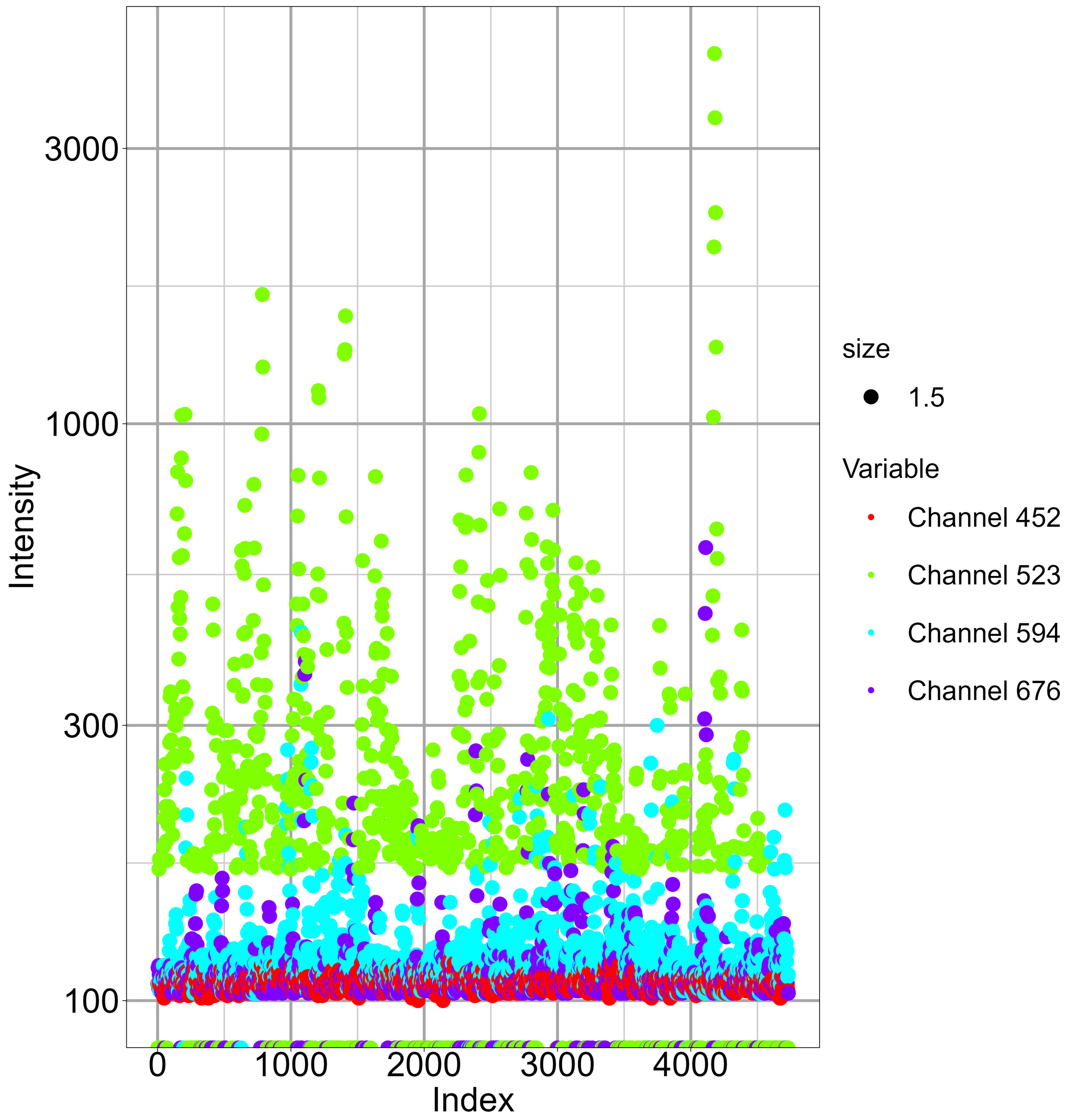

Supplemental Figure 2: Individual Replicates of BrdU Thresholding for Wt, *cds1Δ* and *mrc1Δ*.

SUPPLEMENTAL 2

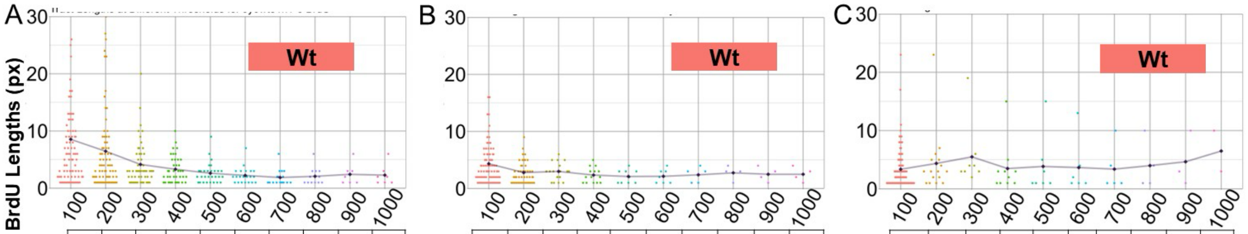

A

n	83	71	51	38	29	21	16	12	7	7
Min.	1	1	1	1	1	1	1	1	1	1
Max.	55	30	20	10	9	7	6	6	6	6
# > 30	4	0	0	0	0	0	0	0	0	0
Avg.	8.5	6.5	4.2	3.3	2.6	2.2	1.9	2.1	2.4	2.3

B

n	75	45	17	14	9	7	5	4	4	4
Min.	1	1	1	1	1	1	1	1	1	1
Max.	16	9	6	5	4	4	4	4	4	4
# > 30	0	0	0	0	0	0	0	0	0	0
Avg.	4.3	2.8	3.0	2.4	2.1	2.1	2.4	2.8	2.5	2.5

C

n	105	16	8	10	7	6	5	4	3	2
Min.	1	1	2	1	1	1	1	1	1	3
Max.	23	23	19	15	15	13	10	10	10	10
# > 30	0	0	0	0	0	0	0	0	0	0
Avg.	3.4	4.4	5.5	3.5	3.9	3.7	3.4	4.0	4.7	6.5

BrdU Thresholding Value →

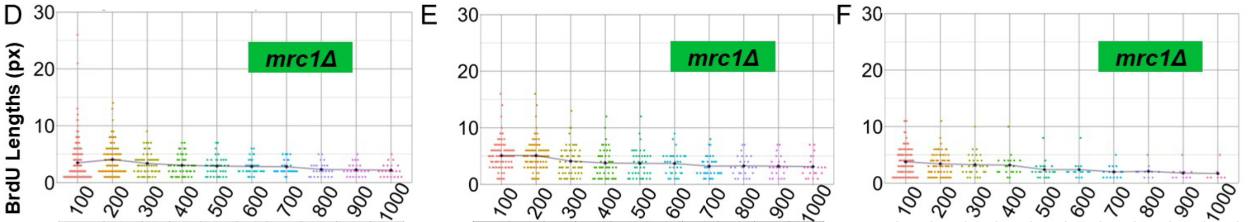

D

n	254	95	58	47	38	31	25	25	23	21
Min.	1	1	1	1	1	1	1	1	1	1
Max.	26	14	9	7	7	6	5	5	5	5
# > 30	0	0	0	0	0	0	0	0	0	0
Avg.	3.5	4.1	3.4	3.0	2.9	2.8	2.8	2.3	2.3	2.2

E

n	80	1	1	1	1	1	1	1	1	1
Min.	16	16	13	12	12	9	8	7	7	7
Max.	0	0	0	0	0	0	0	0	0	0
# > 30	1	1	1	1	1	1	1	1	1	1
Avg.	5.1	5.1	4.1	3.8	3.7	3.7	3.2	3.3	3.2	3.1

F

n	100	63	30	20	20	15	15	10	10	7
Min.	1	1	1	1	1	1	1	1	1	1
Max.	11	11	10	10	8	8	5	5	5	5
# > 30	0	0	0	0	0	0	0	0	0	0
Avg.	3.8	3.4	3.3	3.2	2.4	2.4	2.0	2.1	1.8	1.7

BrdU Thresholding Value →

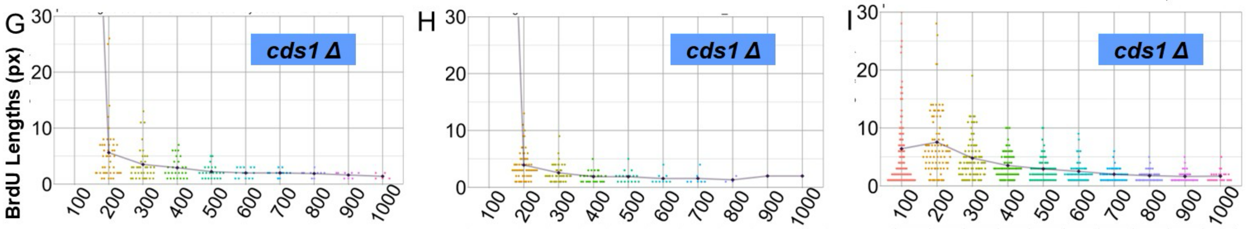

G

n	11	45	37	27	22	14	11	9	8	5
Min.	86	1	1	1	1	1	1	1	1	1
Max.	450	26	13	7	5	3	3	3	2	2
# > 30	11	0	0	0	0	0	0	0	0	0
Avg.	6.4	7.6	4.8	3.5	2.9	2.5	2.0	1.8	1.6	1.7

H

n	11	57	39	28	17	9	7	3	1	1
Min.	64	1	1	1	1	1	1	1	2	2
Max.	427	13	9	5	5	4	4	2	2	2
# > 30	11	0	0	0	0	0	0	0	0	0
Avg.	169.4	3.9	2.6	1.9	1.9	1.6	1.6	1.3	2.0	2.0

I

n	171	85	86	80	66	58	49	44	34	23
Min.	1	1	1	1	1	1	1	1	1	1
Max.	58	35	19	10	10	9	6	5	5	5
# > 30	6	1	0	0	0	0	0	0	0	0
Avg.	6.4	7.6	4.8	3.5	2.9	2.5	2.0	1.8	1.6	1.7

BrdU Thresholding Value →

Supplemental Figure 3: Individual Replicates of Rad51 Thresholding for Wt, *cds1Δ* and *mrc1Δ*.

SUPPLEMENTAL 3

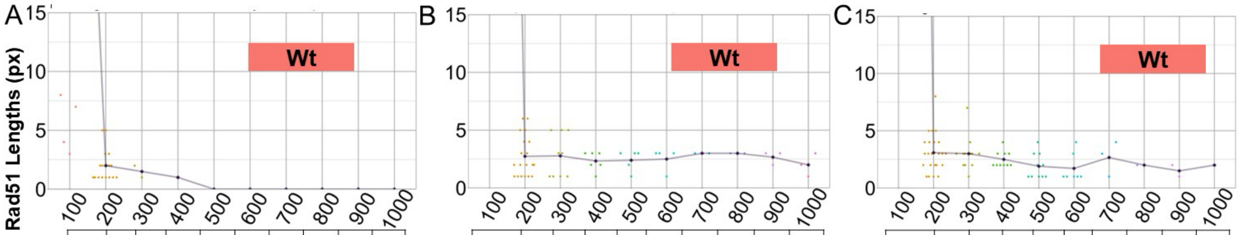

A (Wt)

	100	200	300	400	500	600	700	800	900	1000
n	17	17	2	1	1	1	1	1	1	1
Min.	3	1	1	1	0	0	0	0	0	0
Max.	340	5	2	1	0	0	0	0	0	0
# > 15	13	0	0	0	0	0	0	0	0	0
Avg.	68.6	2.0	1.5	1.0	0.0	0.0	0.0	0.0	0.0	0.0

B (Wt)

	100	200	300	400	500	600	700	800	900	1000
n	7	19	9	6	5	4	3	3	3	3
Min.	72	1	1	1	1	1	3	3	2	1
Max.	280	6	5	3	3	3	3	3	3	3
# > 15	7	0	0	0	0	0	0	0	0	0
Avg.	160.4	2.7	2.8	2.3	2.4	2.5	3.0	3.0	2.7	2.0

C (Wt)

	100	200	300	400	500	600	700	800	900	1000
n	4	23	12	10	10	7	3	2	2	1
Min.	197	1	1	2	1	1	1	2	1	2
Max.	296	8	7	4	4	4	4	2	2	2
# > 15	4	0	0	0	0	0	0	0	0	0
Avg.	230.2	3.1	3.0	2.5	1.9	1.7	2.7	2.0	1.5	2.0

Rad51 Thresholding Value →

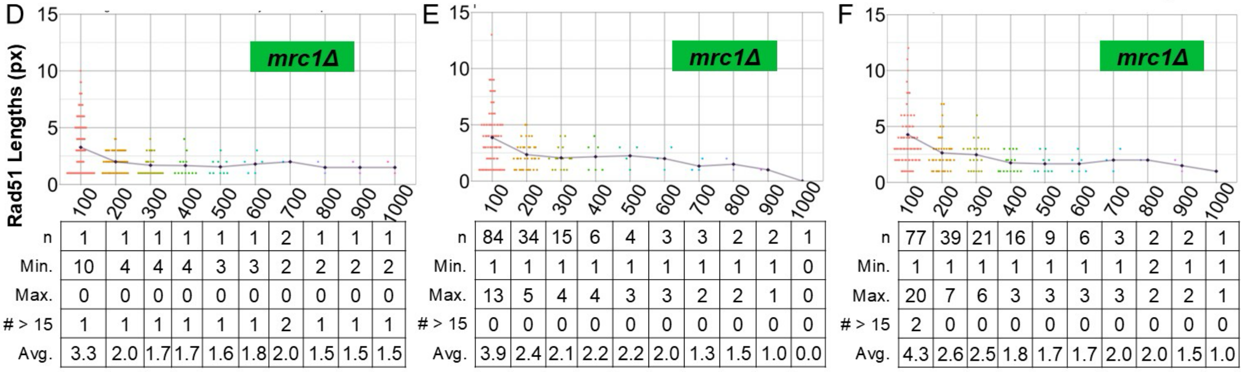

D (*mrc1Δ*)

	100	200	300	400	500	600	700	800	900	1000
n	1	1	1	1	1	1	2	1	1	1
Min.	10	4	4	4	3	3	2	2	2	2
Max.	0	0	0	0	0	0	0	0	0	0
# > 15	1	1	1	1	1	1	2	1	1	1
Avg.	3.3	2.0	1.7	1.7	1.6	1.8	2.0	1.5	1.5	1.5

E (*mrc1Δ*)

	100	200	300	400	500	600	700	800	900	1000
n	84	34	15	6	4	3	3	2	2	1
Min.	1	1	1	1	1	1	1	1	1	0
Max.	13	5	4	4	3	3	2	2	1	0
# > 15	0	0	0	0	0	0	0	0	0	0
Avg.	3.9	2.4	2.1	2.2	2.2	2.0	1.3	1.5	1.0	0.0

F (*mrc1Δ*)

	100	200	300	400	500	600	700	800	900	1000
n	77	39	21	16	9	6	3	2	2	1
Min.	1	1	1	1	1	1	1	2	1	1
Max.	20	7	6	3	3	3	3	2	2	1
# > 15	2	0	0	0	0	0	0	0	0	0
Avg.	4.3	2.6	2.5	1.8	1.7	1.7	2.0	2.0	1.5	1.0

Rad51 Thresholding Value →

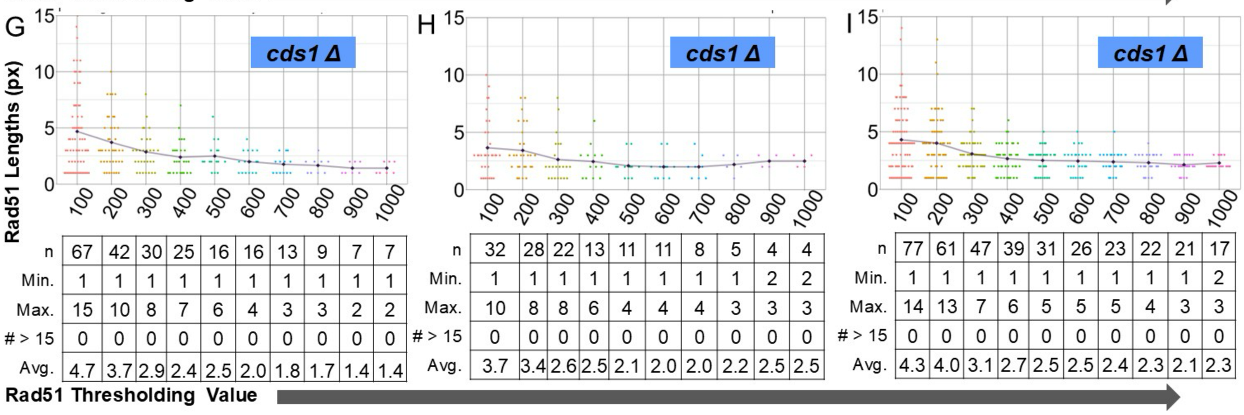

G (*cds1 Δ*)

	100	200	300	400	500	600	700	800	900	1000
n	67	42	30	25	16	16	13	9	7	7
Min.	1	1	1	1	1	1	1	1	1	1
Max.	15	10	8	7	6	4	3	3	2	2
# > 15	0	0	0	0	0	0	0	0	0	0
Avg.	4.7	3.7	2.9	2.4	2.5	2.0	1.8	1.7	1.4	1.4

H (*cds1 Δ*)

	100	200	300	400	500	600	700	800	900	1000
n	32	28	22	13	11	11	8	5	4	4
Min.	1	1	1	1	1	1	1	1	2	2
Max.	10	8	8	6	4	4	4	3	3	3
# > 15	0	0	0	0	0	0	0	0	0	0
Avg.	3.7	3.4	2.6	2.5	2.1	2.0	2.0	2.2	2.5	2.5

I (*cds1 Δ*)

	100	200	300	400	500	600	700	800	900	1000
n	77	61	47	39	31	26	23	22	21	17
Min.	1	1	1	1	1	1	1	1	1	2
Max.	14	13	7	6	5	5	5	4	3	3
# > 15	0	0	0	0	0	0	0	0	0	0
Avg.	4.3	4.0	3.1	2.7	2.5	2.5	2.4	2.3	2.1	2.3

Rad51 Thresholding Value →

Supplemental Figure 4: Individual Replicates of Cdc45 Thresholding for Wt, *cds1Δ* and *mrc1Δ*.

SUPPLEMENTAL 4

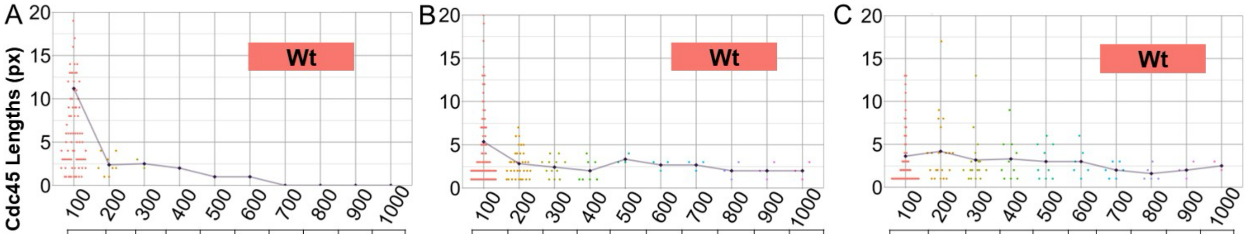

A

n	94	8	2	1	1	1	1	1	1	1
Min.	1	1	2	2	1	1	0	0	0	0
Max.	55	4	3	2	1	1	0	0	0	0
# > 20	18	0	0	0	0	0	0	0	0	0
Avg.	11.2	2.4	2.5	2.0	1.0	1.0	0.0	0.0	0.0	0.0

B

n	139	34	12	8	3	3	3	3	3	3
Min.	1	1	1	1	3	2	2	1	1	1
Max.	59	7	4	4	4	3	3	3	3	3
# > 20	7	0	0	0	0	0	0	0	0	0
Avg.	5.4	2.8	2.4	2.0	3.3	2.7	2.7	2.0	2.0	2.0

C

n	117	21	17	10	9	6	6	5	3	2
Min.	1	1	1	1	1	1	1	1	1	2
Max.	37	17	13	9	6	6	3	3	3	3
# > 20	4	0	0	0	0	0	0	0	0	0
Avg.	3.6	4.2	3.2	3.3	3.0	3.0	2.0	1.6	2.0	2.5

Cdc45 Thresholding Value

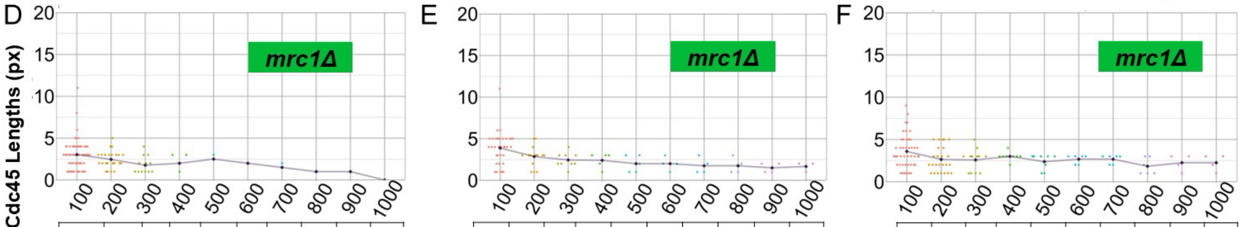

D

n	55	23	12	3	1	1	1	0	0	0
Min.	1	1	1	1	3	2	2	0	0	0
Max.	11	5	4	3	3	2	2	0	0	0
# > 20	0	0	0	0	0	0	0	0	0	0
Avg.	3.0	2.5	1.8	2.0	2.5	2.0	1.5	1.0	1.0	0.0

E

n	34	14	9	5	5	4	4	4	4	3
Min.	1	1	1	1	1	1	1	1	1	1
Max.	11	5	4	3	3	3	3	3	2	2
# >20	0	0	0	0	0	0	0	0	0	0
Avg.	3.9	2.9	2.4	2.4	2.0	2.0	1.8	1.8	1.5	1.7

F

n	44	25	12	8	8	6	6	6	4	4
Min.	1	1	1	2	1	2	2	1	1	1
Max.	9	5	5	4	3	3	3	3	3	3
# > 20	0	0	0	0	0	0	0	0	0	0
Avg.	3.6	2.	2.6	3.0	2.4	2.7	2.7	1.8	2.2	2.2

Cdc45 Thresholding Value

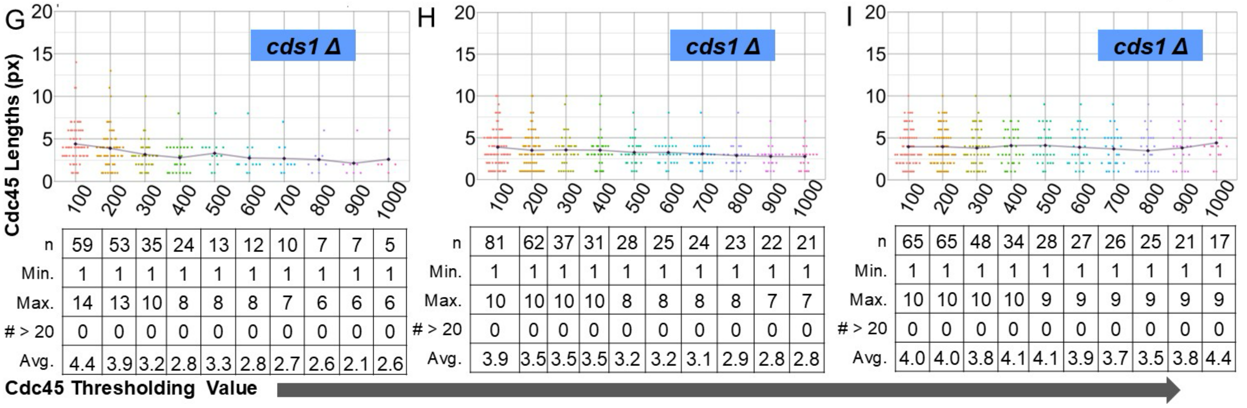

G

n	59	53	35	24	13	12	10	7	7	5
Min.	1	1	1	1	1	1	1	1	1	1
Max.	14	13	10	8	8	8	7	6	6	6
# > 20	0	0	0	0	0	0	0	0	0	0
Avg.	4.4	3.9	3.2	2.8	3.3	2.8	2.7	2.6	2.1	2.6

H

n	81	62	37	31	28	25	24	23	22	21
Min.	1	1	1	1	1	1	1	1	1	1
Max.	10	10	10	10	8	8	8	8	7	7
# > 20	0	0	0	0	0	0	0	0	0	0
Avg.	3.9	3.5	3.5	3.5	3.2	3.2	3.1	2.9	2.8	2.8

I

n	65	65	48	34	28	27	26	25	21	17
Min.	1	1	1	1	1	1	1	1	1	1
Max.	10	10	10	10	9	9	9	9	9	9
# > 20	0	0	0	0	0	0	0	0	0	0
Avg.	4.0	4.0	3.8	4.1	4.1	3.9	3.7	3.5	3.8	4.4

Cdc45 Thresholding Value

Supplemental Figure 5: Individual Replicates of BrdU Smoothing for Wt, *cds1Δ* and *mrc1Δ*.

SUPPLEMENTAL 5

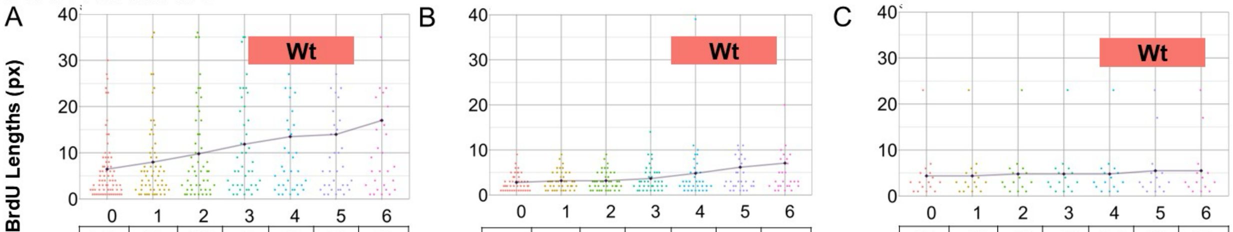

A (Wt)

	0	1	2	3	4	5	6
n	71	59	50	43	39	38	33
Min.	1	1	1	1	1	1	1
Max.	30	36	36	43	51	55	81
# > 40	0	0	0	1	4	4	5
Avg.	6.5	8.0	9.8	11.9	13.5	14.0	17

B (Wt)

	0	1	2	3	4	5	6
n	45	41	41	38	33	29	27
Min.	1	1	1	1	1	1	1
Max.	9	9	9	14	39	56	63
# > 40	0	0	0	0	0	1	1
Avg.	2.8	3.2	3.2	3.7	4.8	6.2	7.1

C (Wt)

	0	1	2	3	4	5	6
n	16	16	15	15	15	14	14
Min.	1	1	1	1	1	1	1
Max.	23	23	23	23	23	23	23
# > 40	0	0	0	0	0	0	0
Avg.	4.4	4.4	4.8	4.8	4.8	5.5	5.5

BrdU Smoothing Value →

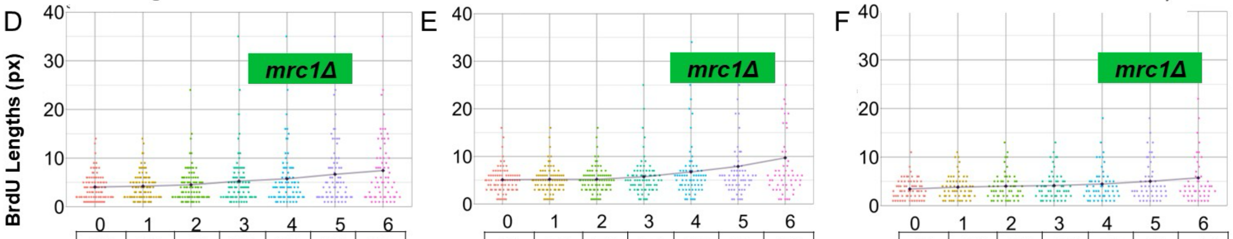

D (*mrc1Δ*)

	0	1	2	3	4	5	6
n	95	92	88	80	76	70	66
Min.	1	1	1	1	1	1	1
Max.	14	14	24	35	35	35	35
# > 40	0	0	0	0	0	0	0
Avg.	4.1	4.2	4.5	5.3	5.8	6.7	7.4

E (*mrc1Δ*)

	0	1	2	3	4	5	6
n	80	79	79	74	67	61	54
Min.	1	1	1	1	1	1	1
Max.	16	16	16	25	34	47	47
# > 40	0	0	0	0	0	2	3
Avg.	5.1	5.2	5.2	5.7	6.7	7.9	9.7

F (*mrc1Δ*)

	0	1	2	3	4	5	6
n	63	58	56	55	53	50	47
Min.	1	1	1	1	1	1	1
Max.	11	11	13	13	18	18	29
# > 40	0	0	0	0	0	0	0
Avg.	3.4	3.8	4.0	4.1	4.5	5.0	5.7

BrdU Smoothing Value →

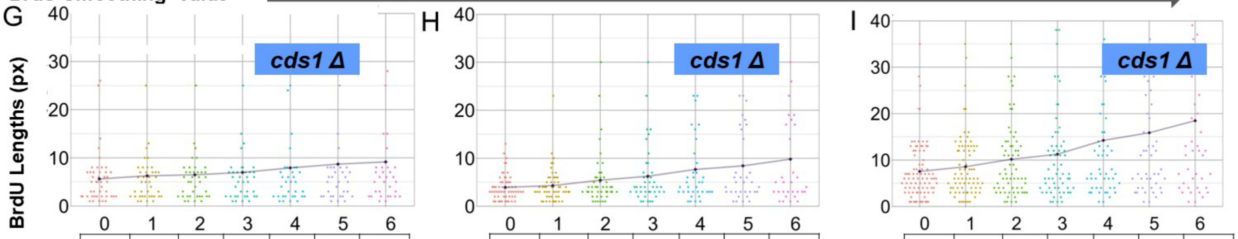

G (*cds1 Δ*)

	0	1	2	3	4	5	6
n	45	41	40	38	35	33	32
Min.	1	1	1	1	1	1	1
Max.	26	41	41	54	54	83	83
# > 40	0	1	1	1	1	1	1
Avg.	5.6	6.3	6.5	7.9	7.9	8.7	9.2

H (*cds1 Δ*)

	0	1	2	3	4	5	6
n	57	53	45	41	36	34	31
Min.	1	1	1	1	1	1	1
Max.	13	23	30	30	30	30	51
# > 40	0	0	0	0	0	0	1
Avg.	3.9	4.3	5.4	6.2	7.7	8.4	9.8

I (*cds1 Δ*)

	0	1	2	3	4	5	6
n	85	76	66	61	51	47	42
Min.	1	1	1	1	1	1	1
Max.	35	35	35	61	77	77	77
# > 40	0	0	0	1	4	4	6
Avg.	7.6	8.6	10.2	11.2	14.2	15.9	18.5

BrdU Smoothing Value →

Supplemental Figure 6: Individual Replicates of Rad51 Smoothing for Wt, *cds1Δ* and *mrc1Δ*.

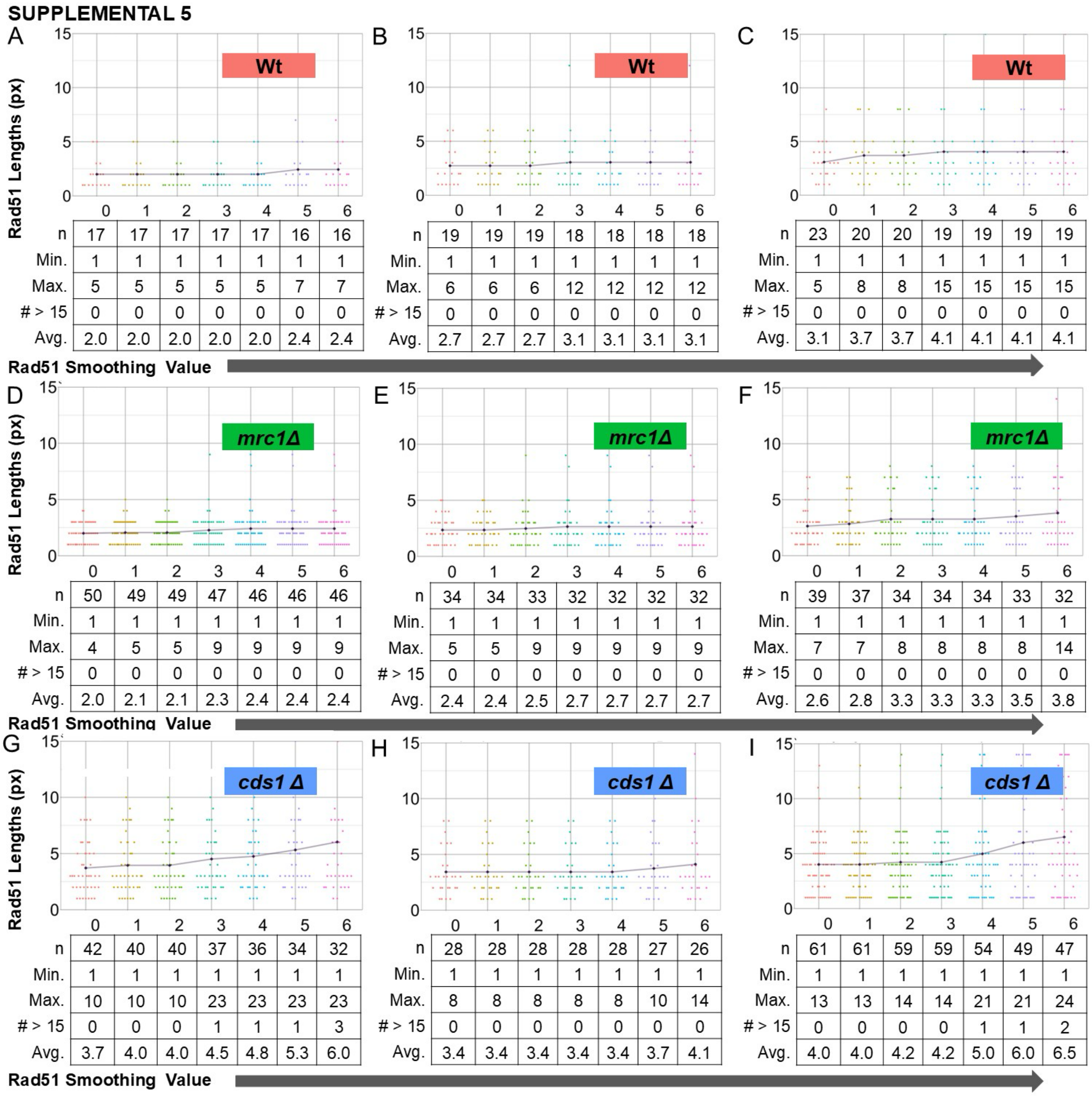

A (Wt)

	0	1	2	3	4	5	6
n	17	17	17	17	17	16	16
Min.	1	1	1	1	1	1	1
Max.	5	5	5	5	5	7	7
# > 15	0	0	0	0	0	0	0
Avg.	2.0	2.0	2.0	2.0	2.0	2.4	2.4

B (Wt)

	0	1	2	3	4	5	6
n	19	19	19	18	18	18	18
Min.	1	1	1	1	1	1	1
Max.	6	6	6	12	12	12	12
# > 15	0	0	0	0	0	0	0
Avg.	2.7	2.7	2.7	3.1	3.1	3.1	3.1

C (Wt)

	0	1	2	3	4	5	6
n	23	20	20	19	19	19	19
Min.	1	1	1	1	1	1	1
Max.	5	8	8	15	15	15	15
# > 15	0	0	0	0	0	0	0
Avg.	3.1	3.7	3.7	4.1	4.1	4.1	4.1

Rad51 Smoothing Value

D (mrc1Δ)

	0	1	2	3	4	5	6
n	50	49	49	47	46	46	46
Min.	1	1	1	1	1	1	1
Max.	4	5	5	9	9	9	9
# > 15	0	0	0	0	0	0	0
Avg.	2.0	2.1	2.1	2.3	2.4	2.4	2.4

E (mrc1Δ)

	0	1	2	3	4	5	6
n	34	34	33	32	32	32	32
Min.	1	1	1	1	1	1	1
Max.	5	5	9	9	9	9	9
# > 15	0	0	0	0	0	0	0
Avg.	2.4	2.4	2.5	2.7	2.7	2.7	2.7

F (mrc1Δ)

	0	1	2	3	4	5	6
n	39	37	34	34	34	33	32
Min.	1	1	1	1	1	1	1
Max.	7	7	8	8	8	8	14
# > 15	0	0	0	0	0	0	0
Avg.	2.6	2.8	3.3	3.3	3.3	3.5	3.8

Rad51 Smoothing Value

G (cds1 Δ)

	0	1	2	3	4	5	6
n	42	40	40	37	36	34	32
Min.	1	1	1	1	1	1	1
Max.	10	10	10	23	23	23	23
# > 15	0	0	0	1	1	1	3
Avg.	3.7	4.0	4.0	4.5	4.8	5.3	6.0

H (cds1 Δ)

	0	1	2	3	4	5	6
n	28	28	28	28	28	27	26
Min.	1	1	1	1	1	1	1
Max.	8	8	8	8	8	10	14
# > 15	0	0	0	0	0	0	0
Avg.	3.4	3.4	3.4	3.4	3.4	3.7	4.1

I (cds1 Δ)

	0	1	2	3	4	5	6
n	61	61	59	59	54	49	47
Min.	1	1	1	1	1	1	1
Max.	13	13	14	14	21	21	24
# > 15	0	0	0	0	1	1	2
Avg.	4.0	4.0	4.2	4.2	5.0	6.0	6.5

Rad51 Smoothing Value

Supplemental Figure 7: Individual Replicates of Cdc45 Smoothing iteration.

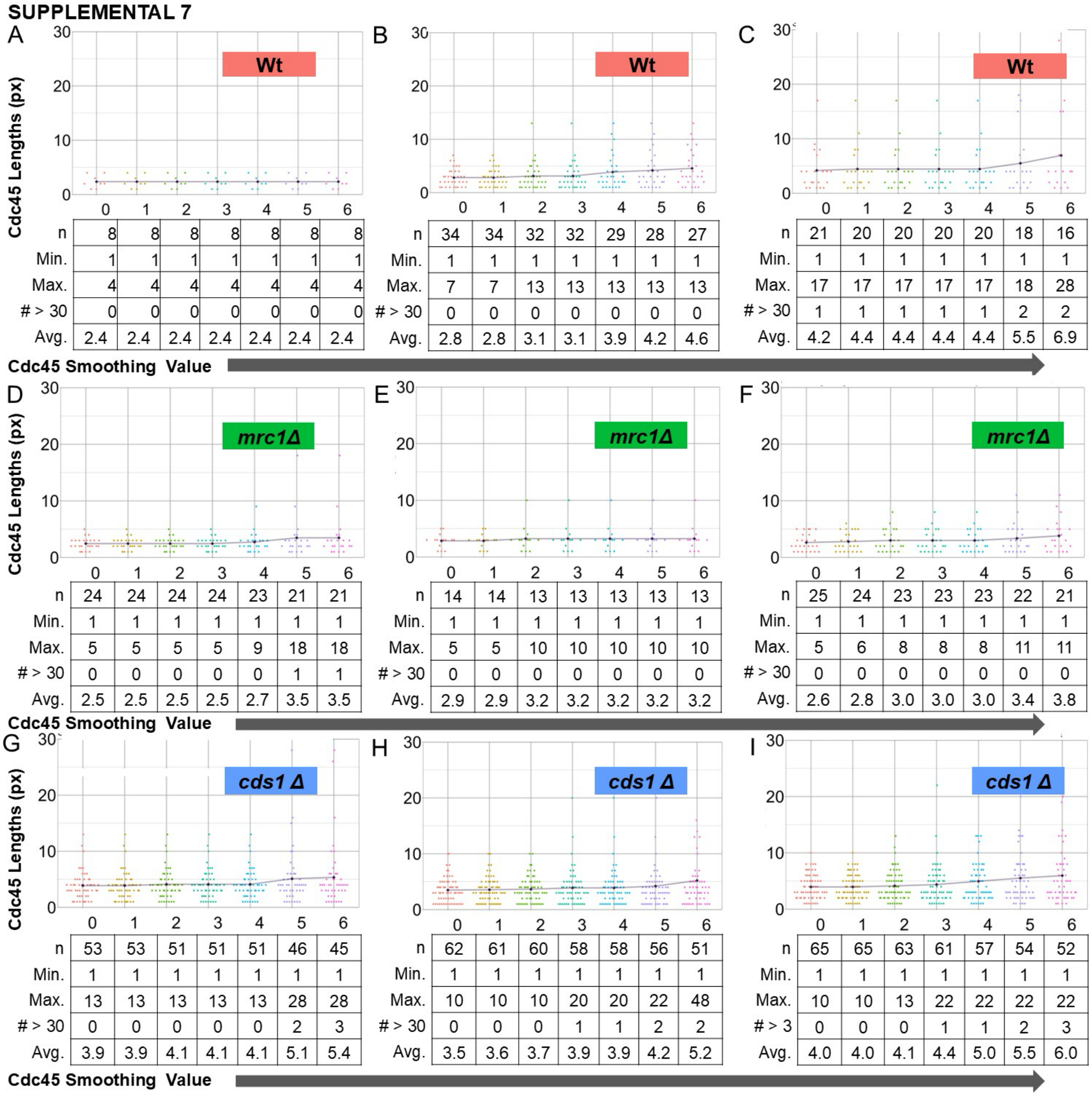

A (Wt)

	0	1	2	3	4	5	6
n	8	8	8	8	8	8	8
Min.	1	1	1	1	1	1	1
Max.	4	4	4	4	4	4	4
# > 30	0	0	0	0	0	0	0
Avg.	2.4	2.4	2.4	2.4	2.4	2.4	2.4

B (Wt)

	0	1	2	3	4	5	6
n	34	34	32	32	29	28	27
Min.	1	1	1	1	1	1	1
Max.	7	7	13	13	13	13	13
# > 30	0	0	0	0	0	0	0
Avg.	2.8	2.8	3.1	3.1	3.9	4.2	4.6

C (Wt)

	0	1	2	3	4	5	6
n	21	20	20	20	20	18	16
Min.	1	1	1	1	1	1	1
Max.	17	17	17	17	17	18	28
# > 30	1	1	1	1	1	2	2
Avg.	4.2	4.4	4.4	4.4	4.4	5.5	6.9

D (*mrc1Δ*)

	0	1	2	3	4	5	6
n	24	24	24	24	23	21	21
Min.	1	1	1	1	1	1	1
Max.	5	5	5	5	9	18	18
# > 30	0	0	0	0	0	1	1
Avg.	2.5	2.5	2.5	2.5	2.7	3.5	3.5

E (*mrc1Δ*)

	0	1	2	3	4	5	6
n	14	14	13	13	13	13	13
Min.	1	1	1	1	1	1	1
Max.	5	5	10	10	10	10	10
# > 30	0	0	0	0	0	0	0
Avg.	2.9	2.9	3.2	3.2	3.2	3.2	3.2

F (*mrc1Δ*)

	0	1	2	3	4	5	6
n	25	24	23	23	23	22	21
Min.	1	1	1	1	1	1	1
Max.	5	6	8	8	8	11	11
# > 30	0	0	0	0	0	0	0
Avg.	2.6	2.8	3.0	3.0	3.0	3.4	3.8

G (*cds1 Δ*)

	0	1	2	3	4	5	6
n	53	53	51	51	51	46	45
Min.	1	1	1	1	1	1	1
Max.	13	13	13	13	13	28	28
# > 30	0	0	0	0	0	2	3
Avg.	3.9	3.9	4.1	4.1	4.1	5.1	5.4

H (*cds1 Δ*)

	0	1	2	3	4	5	6
n	62	61	60	58	58	56	51
Min.	1	1	1	1	1	1	1
Max.	10	10	10	20	20	22	48
# > 30	0	0	0	1	1	2	2
Avg.	3.5	3.6	3.7	3.9	3.9	4.2	5.2

I (*cds1 Δ*)

	0	1	2	3	4	5	6
n	65	65	63	61	57	54	52
Min.	1	1	1	1	1	1	1
Max.	10	10	13	22	22	22	22
# > 3	0	0	0	1	1	2	3
Avg.	4.0	4.0	4.1	4.4	5.0	5.5	6.0

for Wt, *cds1Δ* and *mrc1Δ*.

Supplemental Figure 8: The effect of iterative window sizing on Rad51 and Cdc45 distribution near forks.
A) Rad51 located with forks increases in most in *mrc1Δ*, suggesting that *mrc1Δ* forks accumulate Rad51 that spreads from the fork into unreplicated areas. In contrast, *cds1Δ* forks retain the most Rad51 in unreplicated areas that are away from forks, suggesting DNA damage and more spread away from collapsing forks. From left to right, pixels into the replicated fork area increase from 2 to 10. Extending the number of fork-pixels into replicated area has a small effect on Rad51 localization, regardless of genotype. The 10x10 extreme situation (bottom right) is dominated by the effect of un-replicated pixels made fork-proximal.

B) Expanded window-size effects on Cdc45 colocalization for *cds1Δ (pink), mrc1Δ (green),* and wild type (blue). With no pixels included on either side of the tip, Cdc45 is primarily unreplicated ("COLO UNR REGION") regardless of fork window around BrdU tips. As the grid goes from top to bottom, the number of pixels extending in the unreplicated area near the fork increases from 2 to 10.
